# Borax induces ferroptosis of glioblastoma by targeting HSPA5/NRF2/GPx4/GSH pathways

**DOI:** 10.1111/jcmm.18206

**Published:** 2024-03-17

**Authors:** Cengiz Tuncer, Ceyhan Hacioglu

**Affiliations:** ^1^ Department of Neurosurgery, Faculty of Medicine Düzce University Düzce Turkey; ^2^ Department of Biochemistry, Faculty of Pharmacy Düzce University Düzce Turkey; ^3^ Department of Medical Biochemistry, Faculty of Medicine Düzce University Düzce Turkey

**Keywords:** borax, ferroptosis, glioblastoma multiforme, HSPA5, NRF2

## Abstract

Glioblastoma multiforme (GBM) is a highly aggressive and lethal form of primary brain tumour. Borax has been demonstrated to exhibit anti‐cancer activity through cell death pathways. However, the specific impact of borax on ferroptosis in GBM is not well‐established, and the underlying regulatory mechanisms remain unclear. Initially, the effective concentration of borax on cell viability and proliferation in U251 and A172 cells was determined. Subsequently, the effects of borax on the wound healing were analysed. Nuclear factor erythroid 2‐related factor 2 (NRF2), glutathione peroxidase 4 (GPx4), glutathione (GSH), HSP70 protein 5 (HSPA5), malondialdehyde (MDA) levels and caspase‐3/7 activity were determined in borax‐treated and untreated cells. Finally, the protein expression levels of HSPA5, NRF2 and GPx4 were analysed. Borax suppressed cell viability and proliferation in U251 and A172 cells in a concentration‐ and time‐dependent manner. In addition, borax treatment decreased GPx4, GSH, HSPA5 and NRF2 levels in U251 and A172 cells while increasing MDA levels and caspase‐3/7 activity. Moreover, borax reduced mRNA and protein levels of HSPA5, NRF2 and GPx4 in U251 and A172 cells. Consequently, borax may induce ferroptosis in GBM cells and regulate the associated regulatory mechanisms targeting NRF2 and HSPA5 pathways. This knowledge may contribute to the development of novel therapeutic approaches targeting ferroptosis in GBM and potentially improve patient outcomes.

## 1 INTRODUCTION

Glioblastoma multiforme (GBM) is a highly aggressive and common brain tumour.^1^ It is defined by nuclear abnormalities, cellular variability and rapid cell division. GBM is classified as a grade IV glioma by the World Health Organization and is among the malignant brain tumours, representing a significant cause of mortality in adult patients.^2^ GBM displays cellular variability, spreads extensively throughout the brain, and has a widespread presence, resulting in a grim prognosis for patients. The current standard treatment presently involves surgical removal if possible, followed by radiation therapy and chemotherapy with temozolomide (TMZ).^3^ Nevertheless, the average overall survival time is only 14.6 months, and GBM possesses mechanisms to evade therapeutic interventions by employing various molecular strategies.

Nuclear factor erythroid 2‐related factor 2 (NRF2) is known to play a role in chemotherapy resistance by regulating several antioxidant genes.^4^ Increased NRF2 expression has been associated with resistance to multiple drugs, including cisplatin, doxorubicin, etoposide, and TMZ in cancer cells.^5^ Due to the significance of preventing cell death in drug resistance, promoting controlled cell death has become a promising approach for cancer therapy. Consequently, the induction of ferroptosis has been extensively investigated as a potential therapeutic strategy for treating GBM. The aim is to overcome chemoresistance and improve prognosis in this context, leading researchers to delve deeper into its potential as a therapeutic target.^6^ Ferroptosis is a non‐apoptotic form of cell death that relies on iron and is characterized by the loss of the ability to repair lipid peroxides due to reduced activity of the enzyme glutathione peroxidase 4 (GPx4).^7^ It involves the oxidation of polyunsaturated fatty acids and the accumulation of redox‐active iron. The role of ferroptosis in brain tumours, including GBM, remains an important issue to be studied. Significantly, NRF2 plays a crucial role in regulating various antioxidant genes, including the xc‐ cystine‐glutamate antiporter that facilitates cystine uptake necessary for the synthesis of the antioxidant glutathione (GSH). Additionally, it regulates GPx4, which utilizes GSH as a co‐factor to reduce lipid peroxides and prevent ferroptosis.^8^ As a result, NRF2 expression is closely associated with the modulation of ferroptosis. While NRF2 was initially considered a negative regulator of ferroptosis, inhibiting NRF2 has been found to increase sensitivity to ferroptosis in head and neck cancer cells. Conversely, high expression of NRF2 leads to resistance in glioma cells.^9^ These findings underscore the potential significance of ferroptosis induction in GBM treatment to overcome chemoresistance and provide insights into the role of NRF2 in modulating ferroptosis. However, further research is needed to fully understand the intricate interplay between ferroptosis, NRF2 and drug resistance in brain tumours.

Heat shock proteins (HSPs) are a family of highly conserved proteins expressed in response to physiological and environmental stress, including cancer.^10^ Cancer cells, being under stress, exhibit elevated levels of stress proteins, including chaperones and HSPs. Among the HSP families, the stress‐inducible heat shock protein 70 (HSP70/HSPA) is the most predominant and conserved group.^11^ HSP70 can be found in different parts of the cell and plays various roles in disease processes, such as resistance to therapy and suppression of apoptosis, under different stress conditions. HSP70 protein 5 [HSPA5, also known as glucose‐regulating protein 78 (GRP78)] is highly expressed in many malignant cancers, including gliomas in the central nervous system.^12^ In certain cancers, increased expression of HSPs, like HSPA5, is associated with a poor clinical prognosis and reduced response to treatment. The upregulation of HSPA5 in brain tumours, especially gliomas, has been observed, and its involvement in promoting glioma proliferation, evading apoptosis and facilitating metastatic movement has been identified.^13^ As a result, HSPs are being investigated as potential targets for the development of novel therapeutic agents against GBM. Researchers are exploring strategies to specifically target HSPs, aiming to disrupt their pro‐survival functions and inhibit the progression of GBM. These efforts aim to discover new treatment options that can effectively combat this aggressive form of brain tumour.

In recent years, boron neutron capture therapy (BNCT) has garnered attention as a potential treatment for GBM.^14^ This therapy has demonstrated selective effectiveness in targeting malignant cells while sparing surrounding healthy neurons and blood vessels in the brain. Despite initial concerns about the potential toxicity of boron‐containing compounds, recent studies have shown their non‐toxicity to healthy cells in both in vitro and in vivo experiments. Specific boron compounds, including dihydroxy boronophenylalanine (BPA), sodium borocaptate (BSH), boric acid (BA) and borax, have been utilized in the treatment of malignant melanoma and brain tumours.^15^, ^16^ These findings have debunked misconceptions regarding the toxicity of boron compounds and have generated increased interest in boron‐based therapies.

The study aimed to investigate the potential therapeutic effects of borax on GBM cells by targeting the HSPA5/NRF2/GPx4/GSH signalling pathways involved in regulating ferroptosis. Understanding the association of HSPs and NRF2 with ferroptosis is crucial for comprehending cellular chemoresistance and protection against oxidative stress‐induced cell death. The study focused on examining the effects of borax on HSPA5, NRF2, malondialdehyde (MDA), GSH and GPx4 levels in A172 and U251 malignant glioma cells. By assessing these parameters, we aimed to determine how borax impacts the cellular processes associated with ferroptosis in GBM cells.

## 2 MATERIALS AND METHODS

### 2.1 Cell culture

The U251 and A172 GBM cell lines, as well as SVG cells (human normal brain astroglia cells) used in the study were obtained from the American Type Culture Collection. These cells were cultured in Dulbecco's Modified Eagle medium at 37°C with 5% CO_2_. The culture medium was supplemented with 10% fetal bovine serum (FBS), 100 U/mL Penicillin and 100 μg/mL Streptomycin. Initially, the cells were grown in 25 cm^2^ cell‐culture flasks until they reached confluency. Subsequently, they were transferred to larger 75 cm^2^ cell‐culture flasks. When the cells reached adequate confluency (>80%), the medium was aspirated, and the cells were washed with phosphate buffer solution (PBS)‐EDTA. Finally, the cells were detached using trypsin–EDTA.

### 2.2 Cell viability assay

To assess cell viability, the Enhanced Cell Counting Kit 8 (WST‐8/CCK8, E‐CK‐A362, Elabscience) was utilized. WST‐8 is a compound similar to MTT that can be reduced to orange formazan by mitochondrial dehydrogenases in the presence of an electron coupling reagent. The amount of formazan produced is directly proportional to the number of viable cells. In the experiment, the cells were seeded into 96‐well plates at a density of 5 × 10^3^ cells per well. Once the cells adhered to the bottom of the plates, varying concentrations of borax (ranging from 0 to 800 μM) were added to the wells. The concentration range for borax was determined through preliminary treatments. After specific treatment durations (24, 48 or 72 h), the cell culture media were removed from the wells. The absorbance at 450 nm was measured to indirectly calculate the amount of viable cells. The average viability of untreated cells was considered as 100%, and the viability percentages of the cells treated withs borax were calculated proportionally. The following formula was used to calculate the viability percentages: Cell viability: (OD_treated cells_−OD_blank_)/(OD_control cells_−OD_blank_) × 100.

### 2.3 Cell proliferation analysis

The 5‐bromo‐2′‐deoxyuridine (BrdU) incorporation method is a widely used assay for measuring cell proliferation in vitro. This assay is based on the principle that BrdU is incorporated into the DNA of actively dividing cells during the S phase of the cell cycle. In the experiment, cells were initially cultured until they reached 80% confluence, with a seeding density of 1 × 10^5^ cells per well. The cells were then treated with borax concentrations for 24 h. To assess cell proliferation, the BrdU cell proliferation assay kit (2750, Sigma‐Aldrich) was used following the manufacturer's instructions. This kit allows the detection of BrdU‐incorporated cells using specific antibodies. The absorbance values of the samples were measured at 450 nm, providing quantification of cell proliferation.

### 2.4 Wound‐healing assay

To evaluate the effect of borax (400 μM) on cell migration and wound healing, a confluent monolayer of U251 and A172 cells in a 6‐well plate was subjected to a scratch assay. The scratch was created by dragging a 200 μL pipette tip across the cell monolayer, resulting in a defined wound area. The cells were then washed twice with PBS to remove any debris. To exclude the influence of cell proliferation on the wound healing process, the cells were cultured in medium containing only 1% FBS, which limits cell growth. After creating the wound, the cells were treated with borax. At 0 h (immediately after scratching) and 24 h after treatment, images of the wound area were captured using an Oxion Inverso microscope with a CMEX‐5 Pro camera at 20× magnification. The wound size was quantified using ImageJ software. By comparing the wound area at 0 and 24 h, the extent of wound healing was determined. The wound‐healing percentage was calculated by comparing the reduction in wound area over time and performing statistical analysis.

### 2.5 Cell lysate preparation and biochemical analysis

U251 and A172 cells were seeded at a density of 2 × 10^3^ cells per well in a 96‐well plate. The cells were allowed to grow and form cohesive monolayers before being treated with borax for 24 h. After the treatment period, the cells were washed with PBS to remove any residual borax. The adherent cells were then detached from the plate using trypsin, and the resulting cell pellets were collected in Eppendorf tubes by centrifugation at 1000 × *g* for 5 min at 4°C. To further prepare the cells for biochemical analysis, the cell pellets were washed twice with PBS to remove any remaining traces of trypsin. The pellets were then resuspended in 500 μL radioimmunoprecipitation assay (RIPA) lysis buffer (Santa Cruz Biotechnology, USA). The cell lysates were incubated with RIPA buffer for 20 min at 4°C on an orbital shaker to facilitate cell lysis and extraction of cellular proteins. Subsequently, the cell lysates were centrifuged at 10,000 × *g* for 20 min at 4°C to separate cell debris and other insoluble components from the supernatant. The pellet containing the cell debris was discarded, and the remaining supernatant, referred to as the cell lysate, was collected. The cell lysate was then used for various biochemical analyses to investigate the molecular changes and signalling pathways in the cells following borax treatment.

To measure the levels of HSPA5, NRF2, MDA, GSH and GPx4 in the cell lysates, suitable commercial enzyme‐linked immunosorbent assay (ELISA) kits (EH348RB Invitrogen, MBS764299 MyBioSource, MAK085‐1KT Sigma‐Aldrich, E‐EL‐0026 Elabscience and MBS2000338 MyBioSource, respectively) were utilized. ELISA kits consist of well plates pre‐coated with immobilized antibodies specific to the target proteins. First, the cell lysate samples obtained from the previous steps were added to the respective wells of the ELISA plate, allowing the target proteins to bind to the immobilized antibodies. After incubation and washing steps to remove any unbound substances, specific detection antibodies linked to an enzyme were added to each well. Following another incubation and washing step, a substrate solution that reacts with the enzyme‐linked detection antibody was added. This substrate produces a colour change in proportion to the amount of the target protein present in the sample. The colour intensity was then measured using an ELISA reader (Epoch, BioTek).

### 2.6 Real‐time reverse‐transcriptase polymerase chain reaction (RT‐PCR) for HSPA5, NRF2 and GPx4

Cells were seeded at a density of 3 × 10^5^ cells/well in a 12‐well plate and incubated overnight. The following day, the cells were treated with borax for 24 h. To analyse gene expression levels, total RNA was extracted from both the treated and untreated cell groups using TRIzol® Reagent (Invitrogen) according to the manufacturer's instructions. The extracted RNA was then subjected to reverse transcription using the SuperScript™ IV One‐Step RT‐PCR System (Invitrogen), where 1 μg of RNA from each group was used. For the amplification of complementary DNAs (cDNAs), the StepOnePlus™ Real‐Time PCR System (Thermo Fisher Scientific) was utilized along with SYBR Green Master Mix for RT‐qPCR (Bio‐Rad). Specific primers for HSPA5 (forward: 5′‐CCC GGA CAT TGC CGC AGG‐3′; reverse: 5′‐GCA TGC ATG CGA AGC GGT‐3′), NRF2 (forward: 5′‐GAC CAG TGG ATC TGC CAT‐3′; reverse: 5′‐GCA ACT AGC GGG ATT GCC AGC‐3′), GPx4 (forward: 5′‐AGA GAT CAA AGA GTT CGC CGC‐3′; reverse: 5′‐TCT TCA TCC ACT TCC ACA GCG‐3′) and β‐actin (forward: 5′‐GTG GAC ATC CGC AAA GAC‐3′; reverse: 5′‐AAA GGG TGT AAC GCA ACT A‐3′) were used in the amplification reactions. The PCR cycling conditions consisted of an initial preheat step at 95°C for 10 min, followed by 40 cycles of denaturation at 95°C for 30 s and annealing/extension at 60°C for 1 min. The relative expression of mRNA was determined using the 2^−ΔΔCt^ method, which involves normalizing the gene expression values to the internal control β‐actin.

### 2.7 Western blotting assay

Western blot analysis was performed to assess the protein levels of HSPA5, NRF2 and GPx4 in borax‐treated cells. Initially, protein samples (20 μg) were prepared and separated by sodium dodecyl sulfate polyacrylamide gel electrophoresis (SDS‐PAGE). The separated proteins were then transferred from the gel to a polyvinylidene fluoride (PVDF) membrane. To prevent non‐specific binding, the PVDF membranes were blocked using 2% bovine serum albumin (BSA). Subsequently, the membranes were incubated overnight at 4°C with primary antibodies specific to HSPA5 (dilution 1 μg/mL; PA1‐014A; Invitrogen), NRF2 (dilution 1:2000; PA5‐88084; Invitrogen), GPx4 (dilution 1:1000; PA5‐102521; Invitrogen) and cleaved caspase‐3 (dilution 1:1000; Cat No. 9661; Cell Signaling). Following the primary antibody incubation, the membranes were washed to remove any unbound antibodies. Next, the membranes were incubated with secondary antibodies. To visualize the protein bands, a chemiluminescence ECL (enhanced chemiluminescence) kit (34579, Thermo Scientific) was used. The emitted light was captured using an imaging system, and the resulting images were analysed using software such as ImageJ.

### 2.8 Caspase‐3/7 activation analysis

Caspase‐3/7 activation levels of borax‐treated cells were evaluated using the Muse® Caspase‐3/7 kit (MCH100108; Merck). To determine caspase‐3/7 activation levels, a dead cell marker [7‐aminoactinomycin D (7‐AAD)] was utilized based on the cellular plasma membrane permeability. In the experimental procedure, a total of 1 × 10^5^ cells were incubated with borax for 24 h. After the incubation period, the cells were trypsinized and washed with PBS. Then, 5 μL of the Muse® Caspase‐3/7 working solution was added to 50 μL of the cell suspension. The mixture was incubated at 37°C for 30 min. Subsequently, 150 μL of 7‐AAD was added to the cell medium, and the solution was thoroughly mixed. The final cell suspension was then analysed on the Muse® Cell Analyser.

### 2.9 Statistical analysis

All experiments were performed in triplicate repetitions in three independent experiments. The data obtained from the experiments are presented as the mean values accompanied by mean ± standard deviation (SD). The data were analysed with the Shapiro–Wilk normality test to assess whether they exhibited a normal distribution. To assess the significance of differences between two groups, the Student's *t*‐test was employed. For comparisons among multiple groups, one‐way analysis of variance (ANOVA) and two‐way ANOVA were utilized. All statistical analyses were performed using GraphPad Prism 8. A significance level of *p* < 0.05 (two‐tailed) was chosen as the threshold for determining statistical significance.

## 3 RESULTS

### 3.1 Effects of borax on viability and proliferation in U251, A172 and SVG cells

**FIGURE 1:**
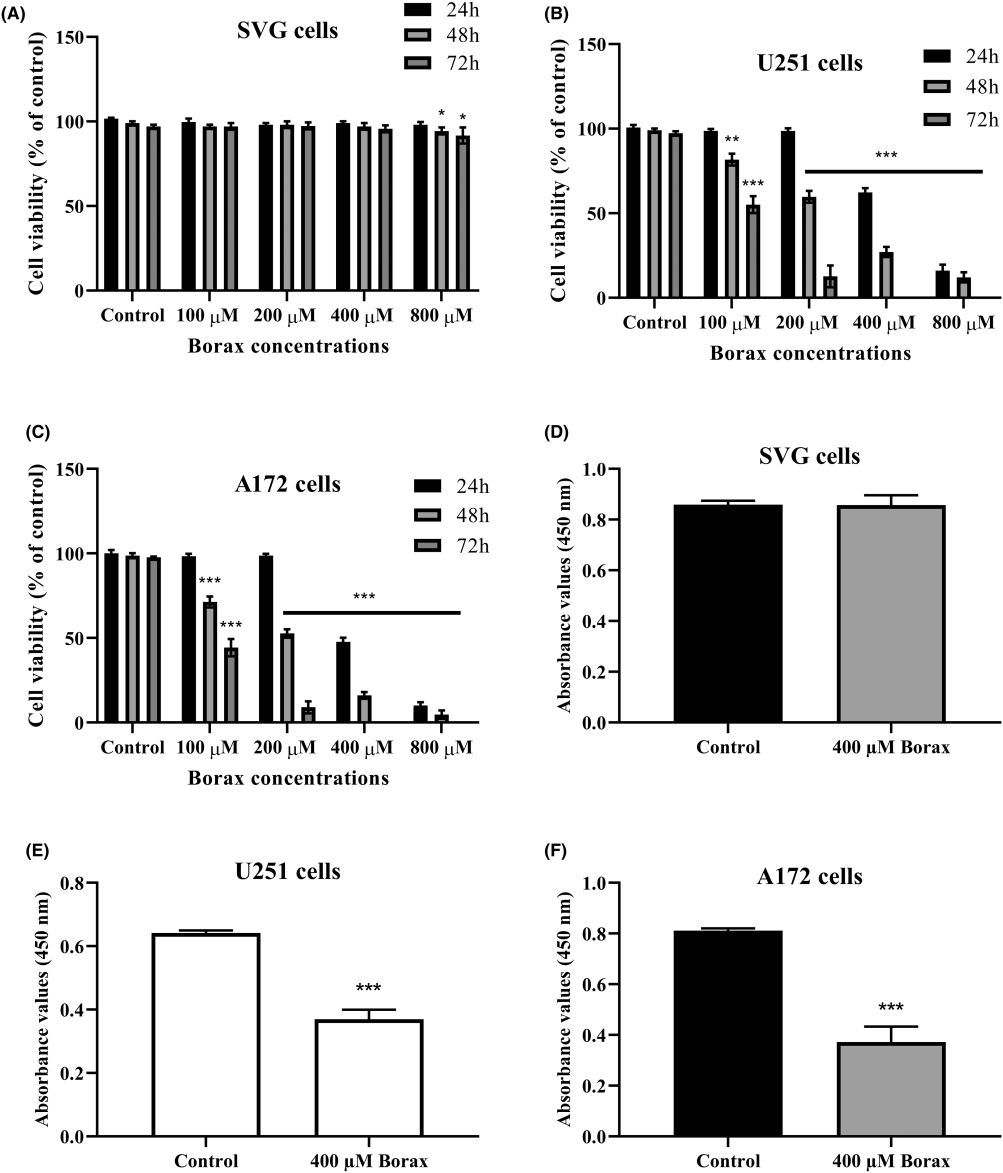
Borax treatment affected cellular viability and proliferation in U251, A172 and SVG cells. (A) CCK8 results in SVG cells; (B) CCK8 results in U251 cells; (C) CCK8 results in A172 cells; (D) BrdU incorporation in SVG cells; (E) BrdU incorporation in U251 cells, (F) BrdU incorporation in A172 cells. All experiments were performed in triplicate repetitions in three independent experiments. *p < 0.05, **p < 0.001 and ***p < 0.0001 versus control group.

Borax treatment resulted in a concentration‐ and time‐dependent reduction in cell viability in U251, A172 and SVG cells (Figure 1). Treating SVG cells with borax concentrations ranging from 0 to 800 μM for 24 h did not result in a significant reduction in viability compared to the control group (Figure 1A). However, after 48 h of exposure to an 800 μM borax concentration, SVG cell viability moderately decreased by 9.6% compared to the control (*p* < 0.05). In addition, treatment with 800 μM borax concentrations for 72 h resulted in a significant reduction (19.1% of control, respectively) in SVG cell viability (*p* < 0.05). CCK8 results showed that there was no significant decrease in cell viability for A172 and U251 cells compared to the control groups after 24‐h treatments with borax concentrations ranging from 0 to 200 μM (*p* > 0.05, Figure 1B,C). In contrast to SVG cells, treatment with borax concentrations of 400 μM and 800 μM for 24 h significantly reduced the viability of U251 (to 41.8% and 85.3% of the control, respectively) and A172 (to 52.5% and 90.8% of the control, respectively) cells compared to the control group. During the 48‐h treatment with 100, 200, 400 and 800 μM borax, U251 cell viability was inhibited by 21.6%, 40.3%, 76.4% and 88.2%, respectively. Similarly, A172 cell viability was suppressed by 30.7%, 47.5%, 84.2% and 95.8%, respectively (*p* < 0.001 and *p* < 0.0001 vs. control). Following a 72‐h treatment period, it was observed that higher borax concentrations (≥400 μM) led to complete inhibition of cell viability in both U251 and A172 cells. Based on the CCK8 data, the IC50 concentrations of borax were determined as 428 μM for U251 cells and 385 μM for A172 cells. This suggests that higher borax concentrations are required to achieve an equivalent level of cell viability inhibition in U251 cells compared to A172 cells. Furthermore, as shown in Figure 1, borax demonstrates greater toxicity towards U251 and A172 cells compared to normal SVG cells, with IC50 values for GBM cells (U251 and A172) ranging between 350 and 450 μM, while SVG cells displayed a higher IC50 value of 2.1 mM (2100 μM). These findings indicate that SVG cells are comparatively less sensitive to the toxic effects of borax when compared to GBM cells. Subsequently, a 400 μM borax concentration was treated for 24 h in U251 and A172 cells during further experimental analyses.

In Figure 1D, it is observed that the treatment of SVG cells with 400 μM borax did not result in a significant inhibition of cell proliferation at 24 h compared to the control. In contrast, 400 μM borax treatment in U251 cells significantly inhibited cell proliferation at 24 h (*p* < 0.0001 vs. control, Figure 1E). Treatment with borax in A172 cells resulted in a significant reduction in proliferation, with a decrease of over 50% compared to the control group (*p* < 0.0001 vs. control, Figure 1F). Furthermore, 400 μM borax exhibited stronger anti‐proliferative effects in A172 cells compared to U251 cells, indicating that A172 cells are more susceptible to the inhibitory effects of borax on cell proliferation. The results indicate that the response to borax treatment varies among different cell types: SVG cells are less affected, U251 cells show moderate sensitivity, while A172 cells displaying a higher degree of susceptibility to the anti‐proliferative effects of borax.

### 3.2 Effects of borax treatment on cell migration rate in U251 and A172 cells

**FIGURE 2:**
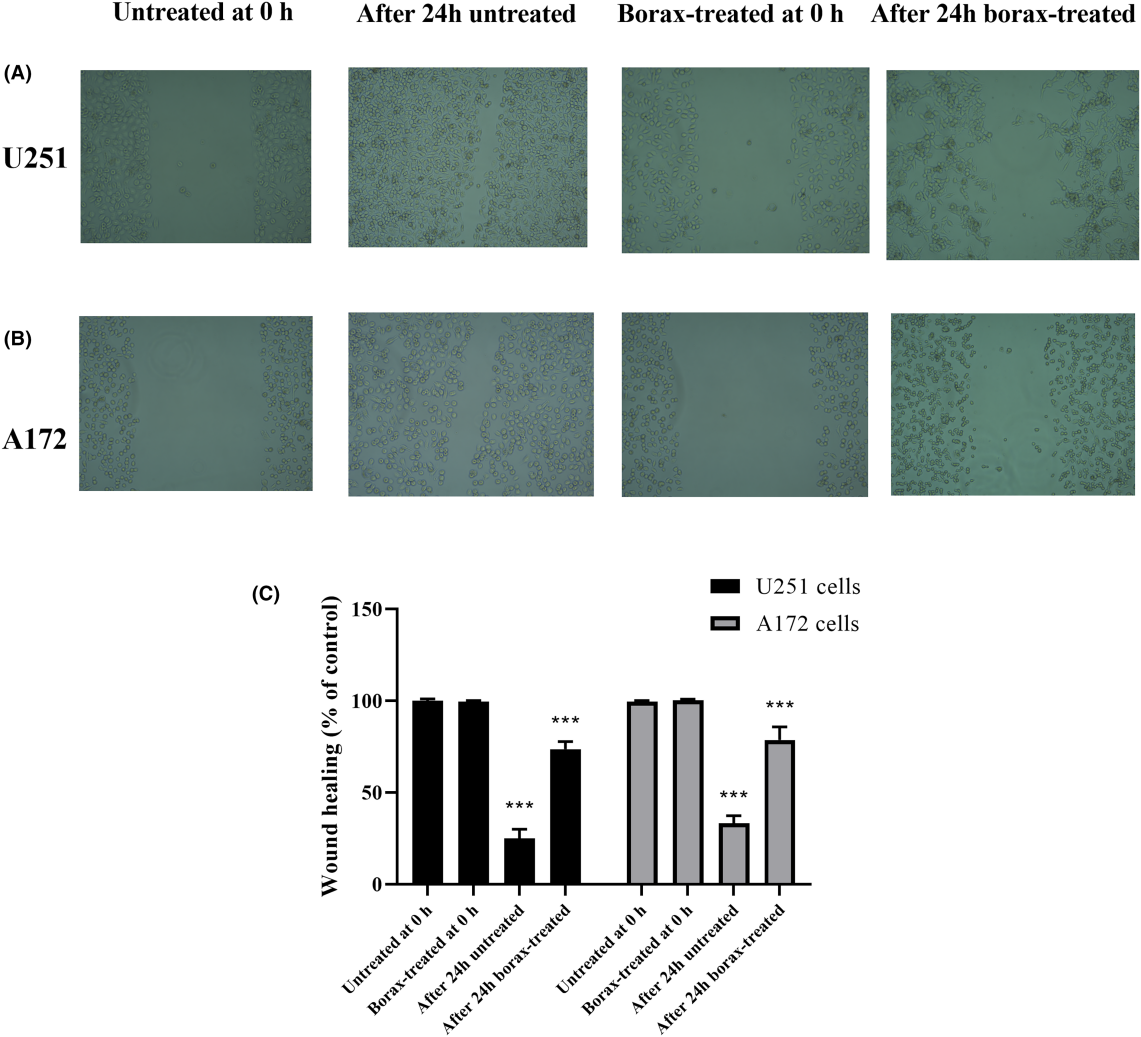
Effects of borax treatment on cell migration rate in U251 and A172 cells. (A) Microscope images of cell migration in U251 cells; (B) microscope images of cell migration in A712 cells; (C) cell migration rate in U251 and A172 cells. All experiments were performed in triplicate repetitions in three independent experiments. ***p < 0.0001 versus untreated group.

The results from the wound‐healing assay (Figure 2A–C) show that 400 μM borax treatment significantly influenced the two‐dimensional migration capacity of both U251 and A172 cells. Without borax treatment, a substantial portion of the wound area closed within 24 h, with U251 cells achieving 85.62% closure and A172 cells achieving 79.25% closure (*p* < 0.0001 vs. untreated cells). However, following treatment with 400 μM borax, both U251 and A172 cells showed a significantly reduced wound‐healing rate. After 24 h, U251 cells closed only 36.22% of the wound area (*p* < 0.001 vs. untreated cells), while A172 cells closed 31.85% (*p* < 0.001 vs. untreated cells). These findings indicate that borax treatment inhibits the migration capacity of both cell types, as evidenced by the reduced closure of the wound area.

### 3.3 Biochemical analysis results of borax treatment in U251 and A172 cells

**FIGURE 3:**
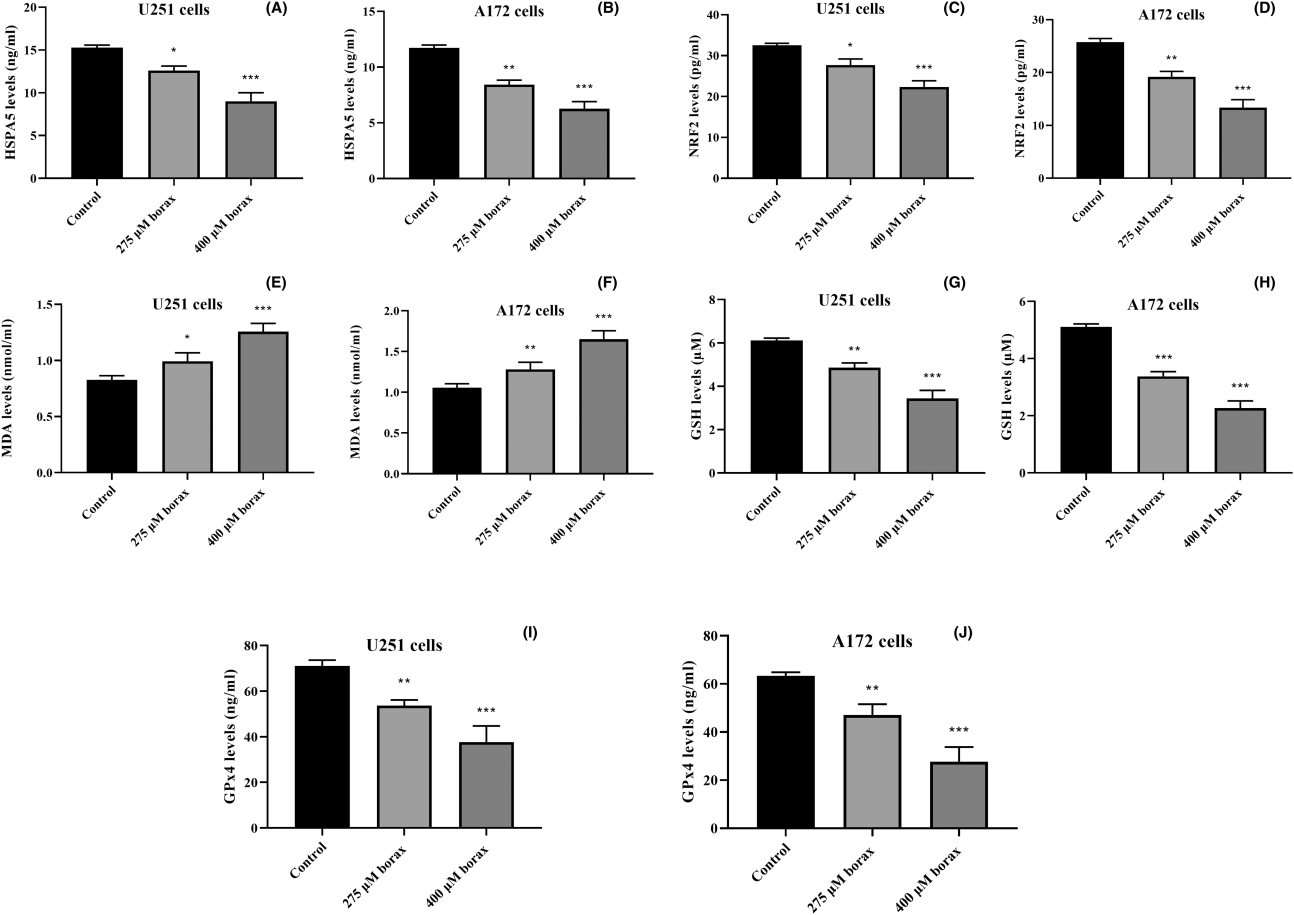
Borax treatment regulated HSPA5, NRF2, MDA, GSH and GPx4 levels in U251 and A172 cells. (A) HSPA5 levels in U251 cells; (B) HSPA5 levels in A172 cells; (C) NRF2 levels in U251 cells; (D) NRF2 levels in A172 cells; (E) MDA levels in U251 cells; (F) MDA levels in A172 cells; (G) GSH levels in U251 cells; (H) GSH levels in A172 cells; (I) GPx4 levels in U251 cells; (J) GPx4 levels in A172 cells. All experiments were performed in triplicate repetitions in three independent experiments. *p < 0.05, **p < 0.001 and ***p < 0.0001 versus control group.

The results in Figure 3 show that borax treatment significantly affected the levels of HSPA5 and NRF2, as well as other factors related to ferroptosis (MDA, GSH and GPx4) in both U251 and A172 GBM cells. In U251 cells (Figure 3A), HSPA5 levels increased by 20.1% and 42.6% when treated with 275 μM (chosen as an intermediate concentration) and 400 μM borax concentrations for 24 h, respectively, compared to the control group (*p* = 0.0013 and *p* < 0.0001). Similarly, in A172 cells (Figure 3B), HSPA5 levels increased by 35.7% at 275 μM and 58.2% at the 400 μM borax concentration compared to the control group (*p* = 0.00052 and *p* < 0.0001).

NRF2 levels in both U251 and A172 cells decreased with increasing borax concentrations. In U251 cells (Figure 3C), treatment with 275 and 400 μM borax concentrations led to a decrease of 15.4% and 37.5% in NRF2 levels, respectively (*p* = 0.0065 and *p* < 0.0001 vs. control). Moreover, in A172 cells (Figure 3D), treatment with 275 and 400 μM borax concentrations resulted in decreases of 26.2% and 52.5% in NRF2 levels, respectively, compared to the control group (*p* = 0.00037 and *p* < 0.0001). This indicates that borax treatment downregulates NRF2 levels in both cell types.

The results from the ELISA analysis demonstrate that borax treatment significantly enhanced MDA levels, a marker of lipid peroxidation, in both U251 and A172 cells. In U251 cells (Figure 3E), MDA levels increased by 20.2% and 45.4% when treated with 275 and 400 μM borax concentrations, respectively, compared to the control group (*p* = 0.026 and *p* < 0.0001). Similarly, in A172 cells (Figure 3F), MDA levels increased by 28.4% at 275 μM and 66.2% at the 400 μM borax concentration compared to the control group (*p* = 0.0028 and *p* < 0.0001).

Furthermore, in U251 cells, GSH levels were reduced by 23.8% at 275 μM and 41.6% at 400 μM borax concentrations compared to the control group (*p* = 0.00018 and *p* < 0.0001; Figure 3G). In addition, A172 cells treated with 275 and 400 μM borax concentrations exhibited decreases in GSH levels by 30.4% and 57.1%, respectively (*p* < 0.0001 vs. control; Figure 3H).

According to the findings shown in Figure 3I, treatment with 275 and 400 μM borax concentrations for 24 h resulted in a reduction of GPx4 levels by 19.4% and 44.7% in U251 cells compared to the control group, respectively (*p* = 0.0066 and *p* < 0.0001). Similarly, in A172 cells (Figure 3J), GPx4 levels decreased by 28.3% at 275 μM and 54.9% at 400 μM borax concentrations (*p* = 0.0024 and *p* < 0.0001 vs. control).

In summary, the results demonstrate that borax treatment induced differential effects on HSPA5, NRF2, GSH and GPx4 levels in A172 cells compared to U251 cells. Specifically, A172 cells exhibited greater reductions in these factors and a more pronounced increase in MDA levels in response to borax treatment. These findings suggest that A172 cells are more sensitive and vulnerable to borax treatment.

### 3.4 Effect of borax on HSPA5, NRF2 and GPx4 expression and protein levels

**FIGURE 4:**
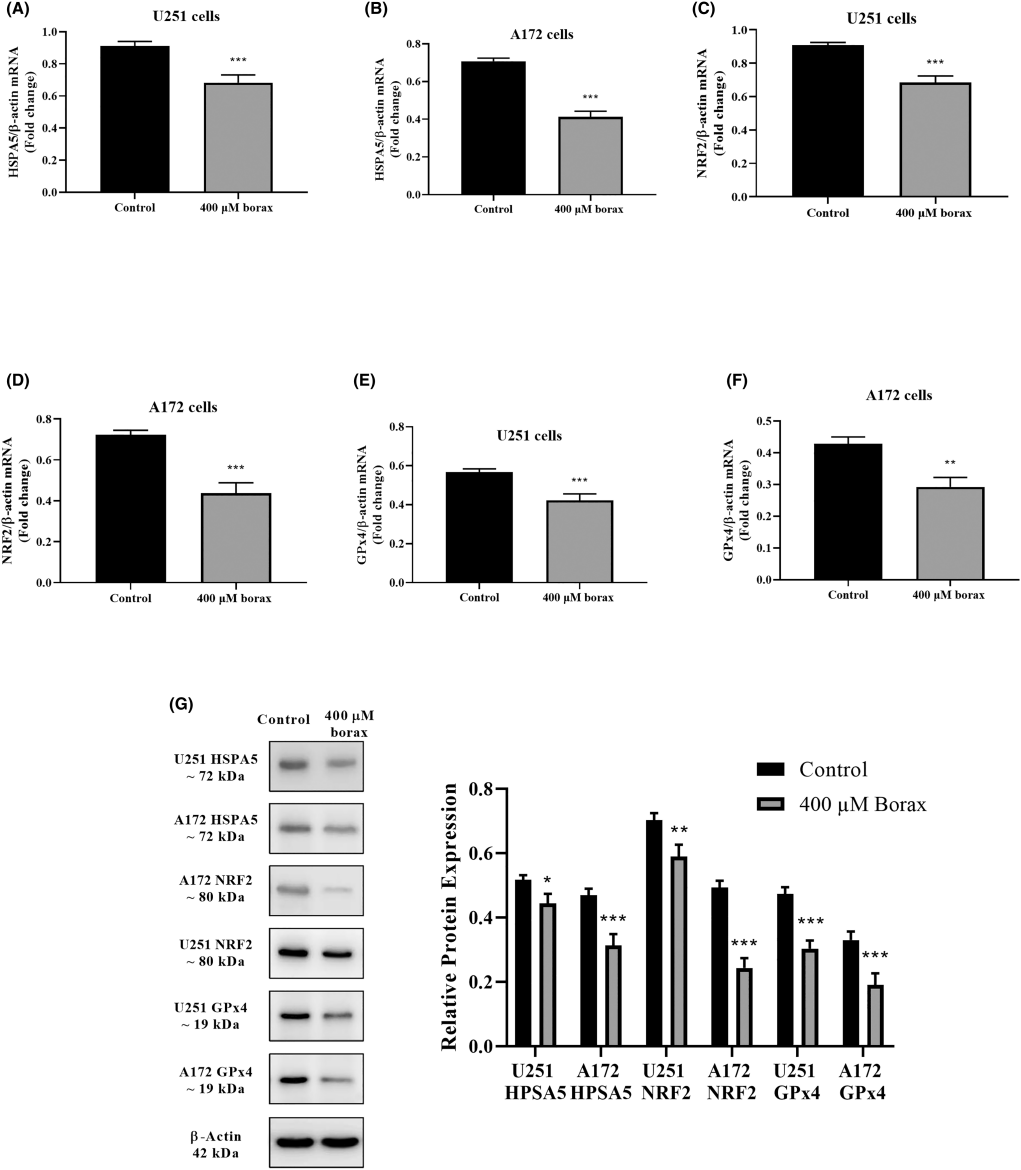
Effects of borax treatment on gene expression and protein levels of HSPA5, NRF2 and GPx4 in U251 and A172 cells. (A) HPSA5 mRNA levels in U251 cells; (B) HSPA5 mRNA levels in A172 cells; (C) NRF2 mRNA levels in U251 cells; (D) NRF2 mRNA levels in A172 cells; (E) GPx4 mRNA levels in U251 cells; (F) GPx4 mRNA levels in A172 cells; (G) relative protein levels. All experiments were performed in triplicate repetitions in three independent experiments.*p < 0.05, **p < 0.01 and ***p < 0.001 versus control group.

The results presented in Figure 4A demonstrate that treatment with 400 μM borax concentrations led to a significant decrease in HSPA5 mRNA levels by 33.7% in U251 cells compared to the control group (*p* < 0.0001). Similarly, in Figure 4B, we can observe a 46.3% decrease in HSPA5 mRNA levels in A172 cells at the 400 μM borax concentration (*p* < 0.0001 vs. control).

In accordance with the information provided in Figure 4C, it is evident that NRF2 mRNA levels in U251 cells exhibited a significant decrease of 29.2% when treated with the 400 μM borax concentration compared to the control group (*p* < 0.0001). Similarly, as shown in Figure 4D, NRF2 mRNA levels in A172 cells decreased by 46.1% when treated with the 400 μM borax concentration (*p* < 0.0001 vs. control).

As for GPx4 mRNA levels, there was a significant decrease in GPx4 mRNA levels in U251 cells treated with the 400 μM borax concentration compared to the control group, showing a 21.8% decrease (*p* < 0.0001; Figure 4E). Similarly, in A172 cells, there was a notable 42.5% decrease in GPx4 mRNA levels when treated with the 400 μM borax concentration (*p* = 0.0016 vs. control; Figure 4F).

Consistent with the results from RT‐PCR and ELISA analyses, Western blot analysis showed that treatment of U251 cells with the 400 μM borax concentration resulted in decreased levels of HSPA5, NRF2 and GPx4 proteins (Figure 4G). Similarly, in A172 cells treated with 400 μM borax, HSPA5, NRF2 and GPx4 protein levels also decreased. Furthermore, borax had a more pronounced suppressive effect on these proteins in A172 cells, consistent with the ELISA results.

### 3.5 Effects of borax on caspase‐3/7 activation in U251 and A172 cells

**FIGURE 5:**
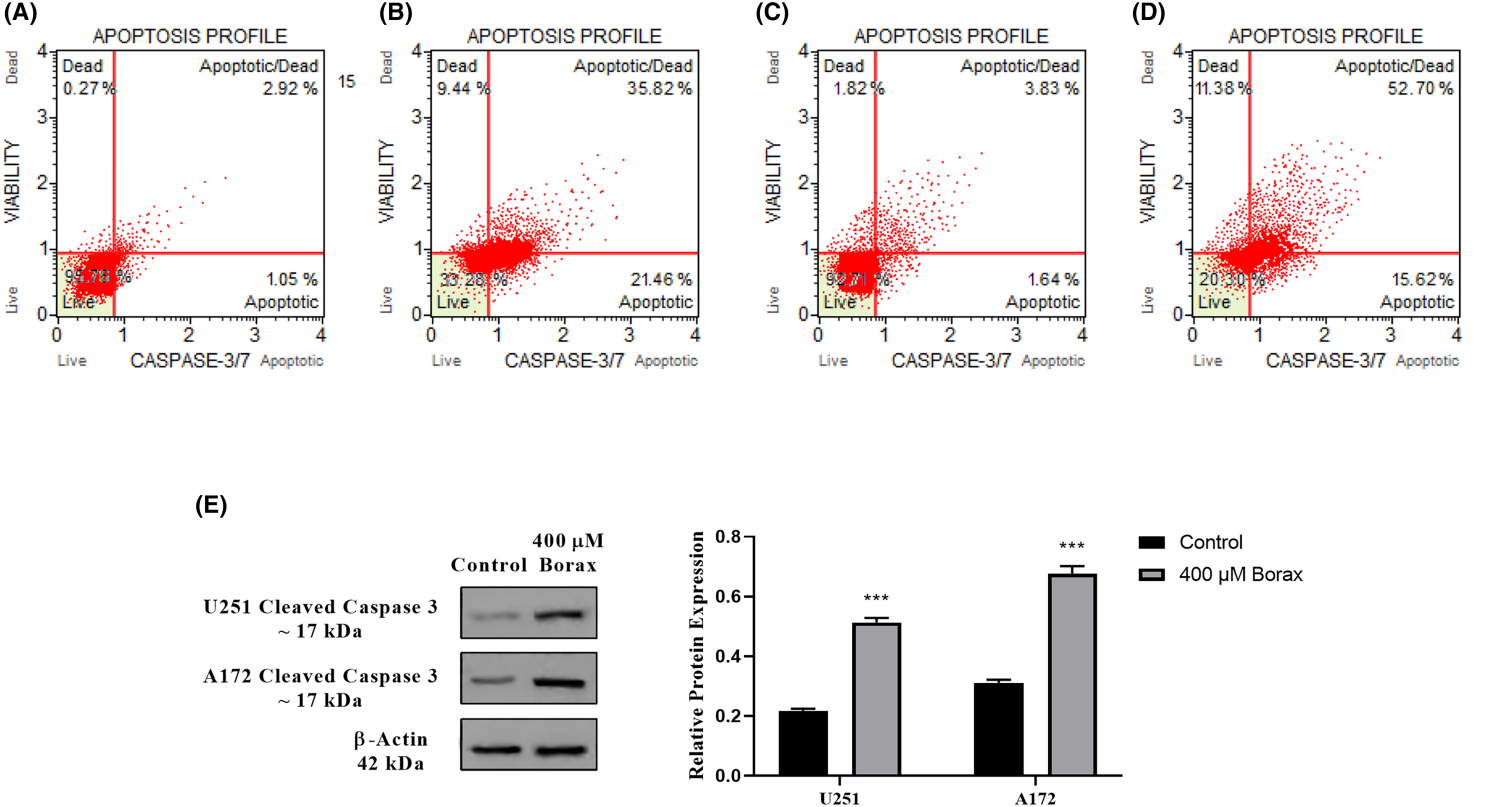
Effects of borax treatment on caspase‐3/7 activation in U251 and A172 cells. (A) Untreated U251 cells group; (B) U251 cells treated with 400 μM borax; (C) untreated A172 cells group; (D) A172 cells treated with 400 μM borax; (E) cleaved caspase‐3 expression levels in U251 and A172 cells treated with 400 μM borax. All experiments were performed in triplicate repetitions in three independent experiments. ***p < 0.001 versus control group.

The effects of borax on the activation of caspase‐3/7 in U251 and A172 cells were shown in Figure 4. In untreated cells, caspase‐3/7 activation was observed at low levels (2.92% in U251 cells and 3.83% in A172 cells) with no significant difference compared to the control group (*p* > 0.05; Figure 5A–C). However, treatment with the 400 μM borax concentration for 24 h significantly enhanced caspase‐3/7 activation in U251 cells, resulting in a 38.82% increase compared to the control group (*p* < 0.0001; Figure 5B). Similarly, in A172 cells treated with the same concentration of borax, caspase‐3/7 activation significantly increased by 52.7% compared to the control group (*p* < 0.0001; Figure 5D).

In line with the findings related to caspase‐3/7 activation, the Western blot analysis revealed that when U251 cells were exposed to a borax concentration of 400 μM, it resulted in higher levels of cleaved caspase‐3 (Figure 5E). Likewise, when A172 cells were treated with 400 μM of borax, there was also an increase in the cleaved caspase‐3 protein levels.

## 4 DISCUSSION

In this study, we investigated the anti‐cancer effects of borax in human GBM cell lines, the U251 and A172 cells. The study revealed novel findings, demonstrating that borax effectively induces cell death in glioma cells through the activation of ferroptosis, a specific form of iron‐dependent cell death characterized by the accumulation of lipid peroxides and the depletion of intracellular antioxidants. Furthermore, our data uncovered that the HSPA5 and NRF2 signalling pathways mediate the effects of borax on glioma cells, playing a crucial role in regulating cellular responses to oxidative stress and serving as key players in ferroptosis regulation. The study also highlighted the inhibitory effect of borax‐induced ferroptosis on cell proliferation in glioma cells, achieved through the modulation of the HSPA5/NRF2/GPx4/GSH regulatory pathways. Additionally, we observed that the reductions in NRF2 and HSPA5 levels caused by borax treatment were consistent with the results of viability, proliferation and migration assays conducted on U251 and A172 cells, suggesting that targeting these specific factors may potentiate the anti‐cancer effects of borax in GBM treatment.

This study assessed the impact of borax, a natural compound, on the viability of human GBM cells. We observed a time‐ and concentration‐dependent reduction in cell viability in both U251 and A172 GBM cell lines following borax treatment, consistent with previous reports demonstrating the cytotoxic effects of borax and boric acid on different cancer cell lines, including U‐87MG cells and DU‐145 human prostate cancer cells.^16^, ^17^ Additionally, our previous study showed that U87‐MG cells exhibited higher BrdU levels compared to HMC3 cells following borax treatment.^18^ This finding suggests that borax treatment had a greater effectiveness and induced more severe cell damage in U87‐MG cells. Boric acid, a boron compound, acts as a mild organic Lewis acid with competitive inhibitory properties, while borax is essentially a salt formed by the combination of a weak acid and a strong base.^19^, ^20^ These unique properties of boron compounds make them promising candidates as anti‐cancer agents because they can influence specific signalling pathways associated with cell death mechanisms in cancer cells.^21^ In recent years, researchers have explored the potential of boron compounds in cancer research. In vivo and in vitro studies have provided evidence of the prophylactic and therapeutic effectiveness of boric acid in rats as well as highlighting its anti‐cancer properties in human endometrial cancer Ishikawa cells and DU‐145 prostate cancer cells.^17^, ^22^, ^23^ Cancer cells are known for producing higher levels of reactive oxygen species (ROS) compared to normal cells, primarily due to disruptions in their redox balance.^24^ This imbalance between antioxidant and oxidant molecules in cancer cells provides an opportunity for cancer therapy.^25^ The objective is to boost ROS production while reducing antioxidant defences, thereby increasing oxidative stress on cancer cells and ultimately triggering pathways leading to cell death. Importantly, our results underscore the significance of evaluating the cytotoxicity of potential anti‐cancer compounds on normal cells to ensure selectivity towards cancer cells. In this regard, we assessed borax's cytotoxicity on normal brain astroglia cells (SVG cells) and found that borax's IC50 values were lower in U251 and A172 cells compared to SVG cells. Additionally, the BrdU assay, which measures cell proliferation, supported the MTT assay results by revealing reduced cell proliferation in U251 and A172 cells treated with borax compared to SVG cells. As previously mentioned, borax treatment demonstrated significantly greater efficacy in cancer cells, particularly in U251 and A172 cells, due to the adjustment of the pro‐oxidant/oxidant balance. These findings suggest that borax exhibits selective cytotoxic effects on GBM cells while causing less damage to normal brain astroglia cells. This selective cytotoxicity is an essential characteristic of potential anti‐cancer compounds, allowing for the targeting of cancer cells while minimizing harm to healthy cells.

NRF2 is a crucial transcription factor that regulates the cellular antioxidant stress response. It interacts with the antioxidant response element (ARE) to control the expression of various antioxidant proteins and enzymes, allowing cells to respond to oxidative stress.^6^ However, the role of NRF2 in tumorigenesis and cancer development is complex. In normal physiological conditions, NRF2 serves a protective role by safeguarding normal cells and inhibiting tumour formation. It plays a vital role in maintaining cellular redox balance and defending against oxidative damage.^26^ However, in the pathophysiological processes of tumorigenesis and cancer progression, NRF2 can exhibit a “dark” side, promoting tumour growth and resistance to treatment.^27^ Researchers have noted that NRF2 and its downstream genes are significantly upregulated in various tumour cells, including gliomas.^28^, ^29^ In this context, NRF2 promotes tumour growth, proliferation and drug resistance. Several studies have specifically investigated the role of NRF2 in TMZ resistance in gliomas. TMZ is a chemotherapy drug commonly used in the treatment of gliomas. Studies have revealed that the treatment of glioma cells with TMZ leads to a notable increase in NRF2 expression.^30^ This heightened NRF2 expression plays a crucial role in contributing to the resistance of glioma cells against TMZ. Conversely, when NRF2 expression is inhibited, it results in heightened sensitivity of glioma cells to TMZ. Consequently, these cells become more vulnerable to the cytotoxic effects of the drug.^31^ Indeed, NRF2 has been shown to play a crucial role in the regulation of ferroptosis. Studies have highlighted the involvement of NRF2 in the modulation of iron homeostasis and the expression of genes related to iron storage and export. By regulating ferroportin 1 and GPx4 genes, NRF2 can influence cellular iron stability and regulate iron‐dependent cell death, including ferroptosis.^32^ Furthermore, NRF2 has been identified as a negative regulator of ferroptosis. For instance, the ferroptosis inducer erastin has been found to inhibit the degradation of NRF2 by modulating the p62‐Keap1‐NRF2 signalling pathway, thereby suppressing ferroptosis in hepatoma cells.^33^ Inhibition of the NRF2‐ARE pathway has been shown to reverse drug resistance and resistance to ferroptosis in head and neck cancer cells.^34^ In the context of GBM, high NRF2 expression has been associated with a poor prognosis and shorter survival time in patients. NRF2 expression was found to be increased threefold in GBM when compared to normal brain tissue.^35^ NRF2 plays a pivotal role in the regulation of ferroptosis by controlling the expression of various genes related to different aspects of cellular function. It governs the expression of genes involved in regulating GSH, including those encoding proteins responsible for GSH synthesis, cysteine supply via xCT, GSH reductase and GPx4. Additionally, NRF2 is involved in the regulation of iron levels by influencing genes associated with iron export and storage, heme synthesis and catabolism.^36^ Moreover, NRF2 also plays a role in NADPH regeneration. Recent research has indicated that NRF2 may partially target xCT, which in turn regulates GPx4 synthesis and function, thus exerting its modulatory effect on ferroptosis.^37^ Inhibition of Kelch‐like ECH‐associated protein 1 (Keap1) activity leads to increased NRF2 activity, resulting in the upregulation of multidrug resistance protein 1 (MRP1), an ATP‐binding cassette (ABC)‐family transporter. MRP1 prevents GSH efflux from cells and significantly inhibits ferroptosis.^38^ When NRF2 is overexpressed, there is a notable increase in xCT mRNA levels, which can reach up to fivefold. This increase in xCT mRNA levels subsequently leads to a reduction in the formation of ROS. On the other hand, when NRF2 expression is low, there is a significant rise in ROS levels.^39^ Consistent with previous studies, this study observed a reduction in NRF2 levels in U251 and A172 cells treated with borax. This reduction in NRF2 levels was accompanied by a decrease in the levels of GSH and GPx4. Notably, the decrease in NRF2 levels after borax treatment led to increased lipid peroxidation, as evidenced by elevated MDA levels. This suggests that borax treatment disrupted the antioxidant defence mechanisms in GBM cells, ultimately leading to the induction of ferroptosis. The results of this study demonstrate that borax treatment in GBM cells leads to the inhibition of GSH synthesis, triggering ferroptosis. Moreover, depletion of GSH alone was sufficient to induce ferroptosis in the cells. This suggests that NRF2's role in ferroptosis may depend on its modulation of intracellular GSH levels following treatment. Furthermore, the higher levels of NRF2, GSH and GPx4 in U251 cells compared to A172 cells are consistent with the protective role of NRF2 in ferroptosis inhibition. The higher expression of NRF2 in U251 cells could contribute to their increased resistance to ferroptosis. NRF2 activation may lead to the upregulation of genes involved in GSH synthesis and GPx4, which are crucial components in the protection against oxidative stress and lipid peroxidation. The observed differences in cell viability, proliferation and caspase‐3/7 activity further support the notion that U251 cells are more resistant to ferroptosis compared to A172 cells. This could be attributed to the higher levels of NRF2, GSH and GPx4 in U251 cells, which contribute to the maintenance of redox homeostasis and the suppression of ferroptosis induction. Indeed, the levels of NRF2 have a direct impact on the sensitivity to ferroptosis. When NRF2 expression is increased, it acts as a protective mechanism and prevents ferroptosis, thereby reducing the vulnerability of cancer cells to this form of cell death. On the other hand, decreased NRF2 expression enhances the sensitivity of cancer cells to ferroptosis, making them more susceptible to this regulated cell death process. Thus, modulating NRF2 expression critically determines cancer cells' fate regarding ferroptosis. These findings highlight the complex role of NRF2 in regulating ferroptosis and its potential implications for targeted cancer therapies.

HSPA5 (also known as GRP78 or binding immunoglobulin protein) is a chaperone protein that plays a crucial role in regulating cell survival and resistance to ferroptosis. This important chaperone protein is often overexpressed in various cancer cells, contributing to the maintenance of protein stability and the promotion of cell viability.^40^ It is induced under conditions of endoplasmic reticulum (ER) stress, including lipid peroxidation, which can contribute to cancer progression and therapy resistance.^41^ While the precise mechanisms are not fully understood, studies have shown that HSPA5 plays a role in modulating ferroptosis in different types of cancer cells. For example, in human pancreatic ductal adenocarcinoma (PDAC) cells, HSPA5 negatively regulates ferroptosis through the HSPA5‐GPx4 signalling pathway, thereby mediating resistance to ferroptosis.^12^ Knockdown of HSPA5 in PDAC cells has been shown to increase lipid ROS levels, such as MDA, and reduce GSH levels, which are characteristic markers of ferroptosis. Erastin, a ferroptosis inducer, can decrease GPx4 protein levels, but HSPA5 has been shown to slow down the degradation process of GPx4 and alleviate ferroptosis.^41^ While the interaction between HSPA5 and GPx4 doesn't completely reverse the erastin‐induced GPx4 decrease, it allows cells more time to adapt to ferroptosis. This potentially enhances cell survival despite eventual ferroptotic cell death. Furthermore, HSPA5 has been reported to maintain the stability of GPx4, a critical enzyme involved in lipid peroxidation and protection against ferroptosis, in glioma cells.^42^ A previous study observed that dihydroartemisinin (DHA) treatment induces the ER stress signalling pathway in U251 cells, specifically resulting in the activation of transcription factor 4 (ATF4) and increased HSPA5 activity.^13^ This activation of ATF4 and the increased activity of HSPA5 in response to DHA‐induced ER stress have been associated with a protective effect against ferroptosis in GBM cells. In the context of DHA‐mediated ferroptosis, the increased activity of HSPA5 plays a crucial role in enhancing the expression and activity of GPx4, an enzyme involved in suppressing lipid peroxidation and protecting against ferroptosis. GPx4 relies on adequate GSH levels and efficient utilization of intracellular iron to perform its function. Consistent with previous studies, our research also revealed an increase in MDA levels in both U251 and A172 cells following treatment with borax. MDA is a well‐known marker of lipid peroxidation, a characteristic hallmark of ferroptosis. Notably, the reduction in HSPA5 levels following borax treatment exacerbated this condition, leading to the production of even higher levels of lipid peroxidation. Consequently, the borax‐treated group exhibited elevated MDA levels, concurrent with a decrease in HSPA5 expression. Furthermore, the reduction of HSPA5 had an adverse impact on GSH levels, a pivotal component of the antioxidant defence system. In this study, borax treatment also affected the expression of GPx4 mRNA, further supporting the notion that borax modulated GPx4 protein stability through HSPA5.

Overall, the study has provided valuable insights into the novel anti‐cancer properties of borax in glioma cells. By elucidating the involvement of the HSPA5 and NRF2 signalling pathways in ferroptosis induction and the inhibition of cell proliferation, our data revealed a previously unknown aspect of borax's mechanism of action. These findings contribute to the understanding of borax as a potential therapeutic agent for GBM treatment and highlight the existence of a negative feedback regulatory pathway in glioma cells. By potentiating the expression and activity of GPx4, HSPA5 regulates lipid peroxidation and the initiation of ferroptotic cell death. The induction of the ferroptotic‐signalling pathway, along with the inhibition of NRF2 and HSPA5, represents a mechanism by which borax‐mediated ferroptosis in GBM cells is modulated cellular viability. One important limitation of our study is that we could not analyse borax for knockdown and overexpression of NRF2 and HSPA5 in primary cell culture and in vivo models. Therefore, it's worth noting that further research is necessary to fully understand the intricate molecular mechanisms involved in the interplay between borax‐induced HSPA5/NRF2/GPx4/GSH in GBM cells.

## AUTHOR CONTRIBUTIONS

**Cengiz Tuncer:** Investigation (supporting). **Ceyhan Hacioglu:** Conceptualization (lead); data curation (lead); formal analysis (lead); funding acquisition (lead); investigation (lead); methodology (lead); project administration (lead); resources (lead); software (lead); supervision (lead); validation (lead); visualization (lead); writing – original draft (lead); writing – review and editing (lead).

## FUNDING INFORMATION

The authors declare that no funds, grants or other support were received during the preparation of this manuscript.

## CONFLICT OF INTEREST STATEMENT

The authors have no relevant financial or non‐financial interests to disclose.

## Data Availability

The data that support the findings of this study are available from the corresponding author upon reasonable request.
